# Systematic Analysis of Pleiotropy in *C. elegans* Early Embryogenesis

**DOI:** 10.1371/journal.pcbi.1000003

**Published:** 2008-02-29

**Authors:** Lihua Zou, Sira Sriswasdi, Brian Ross, Patrycja V. Missiuro, Jun Liu, Hui Ge

**Affiliations:** 1 Department of Statistics, Harvard University, Cambridge, Massachusetts, United States of America; 2 Dana-Farber Cancer Institute, Harvard Medical School, Boston, Massachusetts, United States of America; 3 Whitehead Institute for Biomedical Research, Cambridge, Massachusetts, United States of America; Stanford University, United States of America

## Abstract

Pleiotropy refers to the phenomenon in which a single gene controls several distinct, and seemingly unrelated, phenotypic effects. We use C. elegans early embryogenesis as a model to conduct systematic studies of pleiotropy. We analyze high-throughput RNA interference (RNAi) data from C. elegans and identify “phenotypic signatures”, which are sets of cellular defects indicative of certain biological functions. By matching phenotypic profiles to our identified signatures, we assign genes with complex phenotypic profiles to multiple functional classes. Overall, we observe that pleiotropy occurs extensively among genes involved in early embryogenesis, and a small proportion of these genes are highly pleiotropic. We hypothesize that genes involved in early embryogenesis are organized into partially overlapping functional modules, and that pleiotropic genes represent “connectors” between these modules. In support of this hypothesis, we find that highly pleiotropic genes tend to reside in central positions in protein-protein interaction networks, suggesting that pleiotropic genes act as connecting points between different protein complexes or pathways.

## Author Summary

In a biological system, some genes play single roles while others perform multiple functions. How can we determine which genes are multi-functional? An informative way for probing gene functions is to eliminate the expression of a given gene and observe the phenotypic consequences. RNAi techniques have enabled the generation of genome-wide phenotypic data. Conventionally, genes are clustered into mutually exclusive categories according to the observed defects following RNAi. However, assigning genes that may play multiple roles exclusively into a single category is arbitrary. This paper works out a computational approach that categorizes genes while allowing assignment of genes with complex phenotypes into multiple categories. We apply this approach to genes involved in cell divisions of *C. elegans* early embryos, and find that about half of these genes can be assigned to more than one functional category. This approach has allowed the identification of previously undiscovered gene functions. We also find that genes playing many roles in early embryos tend to reside in central positions in protein networks. Our approach can be used to perform functional annotations based on phenotypic data in other systems and to identify genes that coordinate multiple biological functions.

## Introduction

The phenomenon of pleiotropy highlights the fact that some genes in the genome perform multiple biological functions. Although individual examples of pleiotropic genes have been discovered [1]–[4], pleiotropy remains a poorly understood genetic phenomenon and there have been very few systematic studies. In *S. cerevisiae*, the collection of mutant strains for nearly all genes has enabled high-throughput tests of growth fitness under a variety of environmental conditions [5],[6]. The degree of pleiotropy has been estimated based on the number of conditions under which mutant strains showed abnormal fitness [6]. In multi-cellular organisms, the availability of high-throughput RNAi techniques may lead to the opportunity for systematic analysis of pleiotropic genes. However, when multiple phenotypic effects are present, it is not obvious whether the phenotypic effects should be attributed to the loss of a single function or to multiple functions. For example, a phenotypic effect at earlier stages of animal development may accumulate during cell divisions and migrations, resulting in many defects at later stages of development. In this case, although many defects are observed, they can all be accounted for by the loss of a uniform gene function. Therefore, it is not clear how pleiotropic genes should be identified in practice and what mechanisms lie behind pleiotropy.

*C. elegans* is especially amenable to genome-wide loss-of-function analyses because of well-characterized anatomy, short life cycle, and the convenience of RNAi techniques. The *C. elegans* early embryo is a model system for studying mitotic cell divisions. Piano *et al* screened a set of ovary-enriched genes by RNAi and systematically described early embryonic defects for 161 genes in terms of RNAi-associated phenotypes [7]. Using the RNAi data, they grouped these genes into “phenoclusters”, which correlated well with functional annotations of these genes. Sonnichsen et al. performed whole-genome RNAi experiments to search for genes involved in early embryogenesis [8]. They defined a series of cellular defects occurring in the first two cell divisions, and identified 661 genes that showed at least one of these defects. These genes were manually grouped into functional classes. For example, genes involved in cell polarity were grouped together since the RNAi of these genes resulted in symmetric cell divisions; genes involved in DNA damage checkpoints were grouped together since the RNAi of these genes resulted in delayed P_1_ cell division. Multiple defects during early cell divisions can be scored when a single gene is perturbed. All the scored defects happen in the first approximately 50 minutes of embryonic development, up to a four-cell stage embryo. This short time window ensures that most observed defects are direct rather than secondary. These data and information provide an excellent biological context to systematically explore the phenomenon of pleiotropy.

In this paper, we address several open questions regarding pleiotropy using *C. elegans* early embryogenesis as the model system. First, how can complex phenotypes be decomposed and be linked to the loss of specific biological functions? Second, how can we systematically identify pleiotropic genes? Third, does pleiotropy exist commonly in a biological system? Finally, what potential mechanisms underlie pleiotropy? We find that sets of cellular defects (or “signatures”) are well correlated with losses of certain biological functions, and these signatures can be used to decompose complex phenotypic profiles so as to provide functional annotations. Approximately half of the genes involved in early embryogenesis are found to be pleiotropic, suggesting the prevalence of pleiotropy in biological systems. By integrating phenotypic profiles with protein-protein interaction networks, we observe that highly pleiotropic genes tend to show a higher network “betweenness” [9] than other genes involved in early embryogenesis, suggesting that pleiotropic genes play an important role in connecting various biological pathways.

## Results

### Phenotypic Profiles

**Figure 1:**
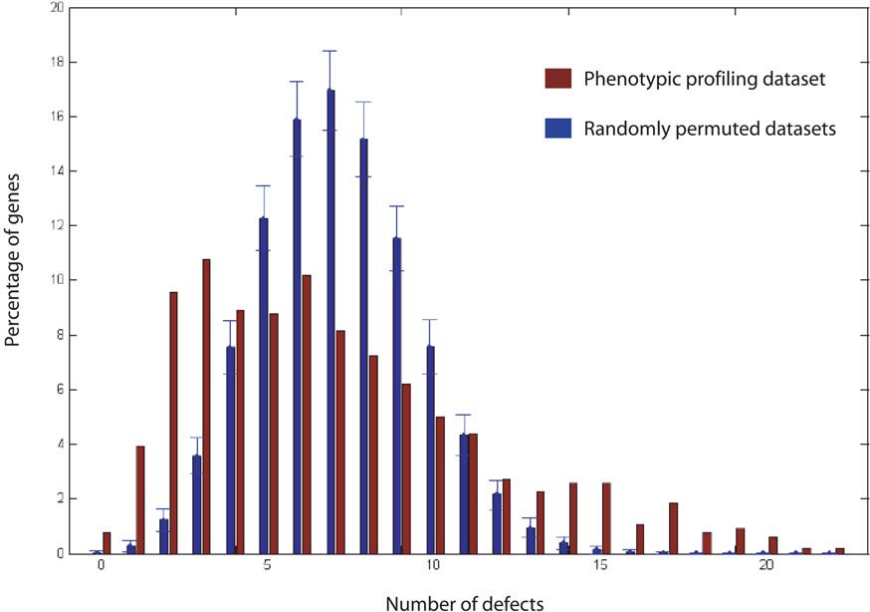
The distribution of the percentage of genes against the number of defects in their phenotypic profiles. We plot the distribution of the percentage of genes against the number of defects (brown bars) and compare with that of randomly permuted datasets (blue bars). The error bars show the standard deviation of the percentages of genes in the randomly permuted datasets. On average, genes in the dataset show 7 cellular defects in their phenotypic profiles. About 10% of the genes show 15 or more defects, much higher than that of the randomly permuted dataset.

Systematic RNAi screens have identified genes involved in early embryogenesis and have characterized their phenotypic profiles, which are composed of a series of cellular defects [8]. As has been described previously [8], phenotypic data can be visualized in a matrix where rows index genes and columns index defects. A gene is given a score of either zero (absence) or a positive value (presence) for each of the 45 defects [8]. We plotted the distribution of the percentage of genes involved in early embryogenesis against the number of defects for which the genes have positive scores (Figure 1). By randomly permuting the values among genes while keeping each column sum fixed (i.e., fixing the total number of genes each defect is associated with), we generated random control datasets and observed that significantly more genes in the real data set exhibit a large number of loss-of-function defects than those in random control sets. In the real dataset, 57 out of 661 genes show 15 or more defects, whereas on average only 1 gene is expected to show this number of defects in a randomly permuted dataset (*P*-value<0.001, see Methods).

### Correlation among Cellular Defects

Genes exhibiting a large number of defects in their phenotypic profiles may be candidates for pleiotropic genes. However, should the degree of pleiotropy be solely determined by the number of defects? It is possible that occurrences of some cellular defects are highly correlated with one another. The highly correlated defects are likely caused by the perturbation of a single-function gene rather than a pleiotropic gene.

**Figure 2:**
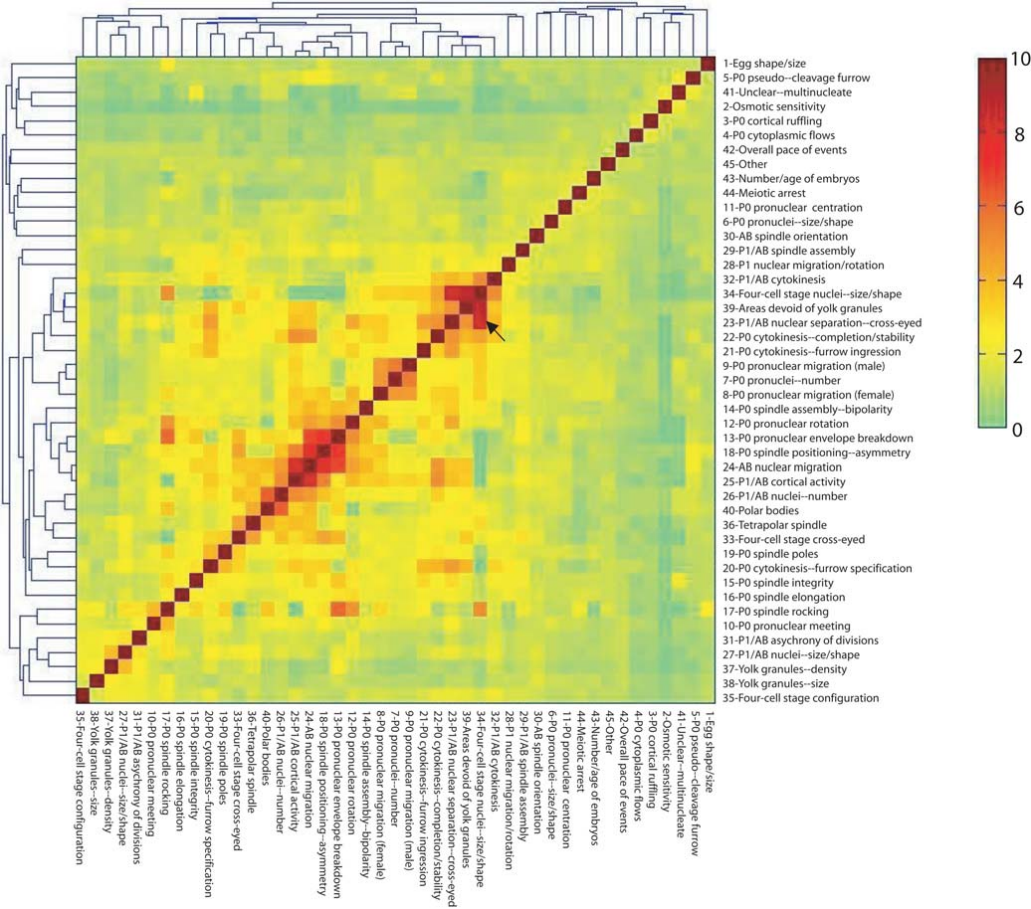
A correlation map for pairs of defects involved in *C. elegans* early embryogenesis. We calculate the ratio of observed co-occurrence to the expected co-occurrence for every pair-wise combination of defects and plot the ratios into a correlation map. A ratio that is higher than 1 indicates the two defects are more likely to co-occur than expected by chance. Some defects, such as *P_1_/AB nuclear separation—cross-eyed* and *four-cell stage nuclei—size/shape* (pointed to with a black arrow), co-occur at a very high frequency. In this map, the co-occurring defects are grouped together by hierarchical clustering.

In order to investigate how strongly cellular defects correlate with each other, we analyzed the occurrence of each individual defect and the co-occurrence of each pair of defects. We then computed the ratio of the observed co-occurrence of each defect pair to the expected co-occurrence as if the two defects occurred independently (see Methods). We plotted the ratios as a correlation map (Figure 2) and found that some defects co-occur much more frequently than expected, while some never co-occur in the same phenotypic profile, suggesting that not all defects occur independently from each other. For example, *P_1_/AB nuclear separation—cross-eyed* (Defect 23) and *four-cell stage nuclei—size/shape* (Defect 34) co-occur at very high frequency, suggesting that embryos showing defects in nuclear separation at the two-cell stage are very likely to be abnormal in nuclear size and shape at the four-cell stage. *P_1_/AB nuclear separation—cross-eyed* also co-occurs with *P_0 _cytokinesis—furrow specification* (Defect 20) and several other defects, and *four-cell stage nuclei—size/shape* also co-occurs with *P_0 _spindle rocking* (Defect 17) and several other defects.

**Figure 3:**
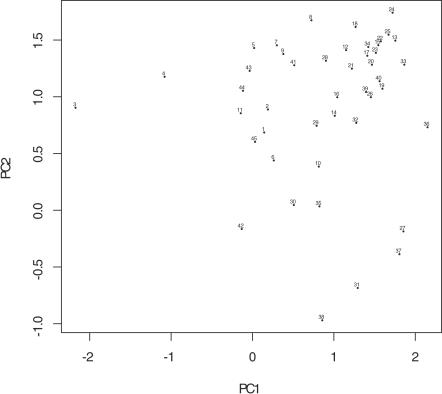
LPCA analysis of the cellular defects. The X axis and Y axis represent the first and the second principal components in LPCA analysis, respectively. The data points labeled with numbers represent the cellular defects, which can be separated according to first two components. The correspondence between numbers and defects are the same as in Figure 2. The defects in proximity in the graph are likely to be closely related biologically.

We also analyzed the occurrence of cellular defects by both linear principal component analysis (PCA) and logistic principal component analysis (LPCA) [10]. Although LPCA appears to be more appropriate for 0-1 type of data, PCA is more appealing in terms of its interpretability because the dimensions of LPCA are not orthogonal and the eigenvalues of LPCA cannot be used to rank the importance of principle components. As dimensional reduction tools, both PCA and LPCA gave similar results for this dataset–the projection of the defects onto the plane spanned by the first and second principal components (PCs) reveals very similar pattern (Figure 3, for LPCA). For example, *P_0 _cytokinesis—furrow specification* (Defect 20), *P_1_/AB nuclear separation—cross-eyed* (Defect 23), *four-cell stage nuclei—size/shape* (Defect 34), and *P_0 _spindle rocking* (Defect 17) show high co-occurrence in the correlation map, and they are positioned close to one another in the LPCA plot as well. The observation of closely related defects suggests that the degree of pleiotropy cannot be readily measured by simply counting the number of defects. In order to study pleiotropy, we need to identify combinations of defects, or “phenotypic signatures,” which describe the effects of losing individual biological functions.

### Phenotypic Signatures

**Figure 4:**
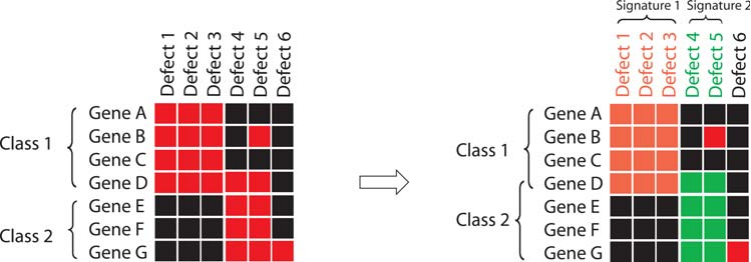
A scheme of our method for identifying phenotypic signatures. We start with pre-defined classes and search for defects that are enriched in each class. The enriched defects compose the phenotypic signature of a given class. We then search for genes that were originally not included in the class but can be matched with the phenotypic signature the class. In this process, a gene may be assigned to multiple classes. The defects shown in orange and green represent phenotypic signatures of two different classes. In this example, Gene D is re-assigned to both classes.

Cell divisions in early embryogenesis involve a number of biological functions such as chromosome segregation, cytokinesis, and cell polarity. Sonnichsen et al. manually grouped genes identified in the RNAi screen into 23 mutually exclusive classes according to their phenotypic profiles [8]. Among these, 22 classes have functional annotations and the remaining one is composed of genes whose phenotypic profiles contain a large number of defects and do not resemble profiles of any functionally characterized genes. We designed a computational approach to determine phenotypic signatures for each of the 22 functional classes and to identify additional genes potentially belonging to the given class (Figure 4).

The phenotypic signature of a class is defined as a collection of cellular defects significantly enriched in that class as compared to the whole dataset. More specifically, for each class as defined in [8], we computed the *P*-value for the enrichment of each defect according to the hypergeometric distribution. This class' phenotypic signature is then composed of all defects whose enrichment *P*-values are no greater than 0.05 after correcting for multiple comparisons. As a result, we found phenotypic signatures for 18 of the 22 functional classes. For the remaining 4 classes, no significantly enriched defects could be identified, because these classes all contained too few genes (5 or fewer) for any defect to pass our statistical threshold.

**Figure 5:**
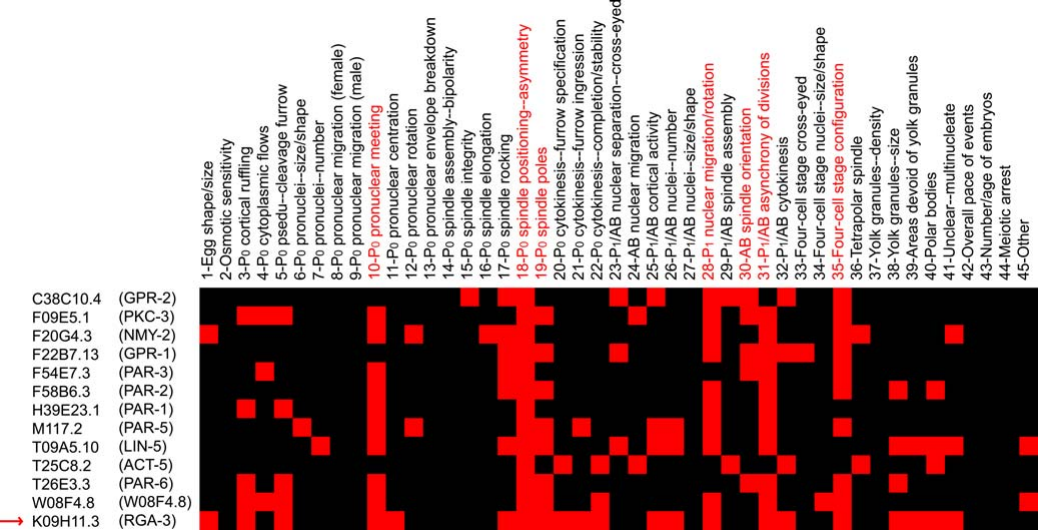
The phenotypic signature for the *cell polarity* class. This signature consists of 7 defects (highlighted in red). One additional gene, K09H11.3 (RGA-3) (pointed to by a red arrow), is assigned to the *cell polarity* class.

The above procedure can be illustrated for the *cell polarity* class (Figure 5). Originally, a total of 12 genes, including some genes previously known to be involved in cell polarity, were assigned to this class. We identified 7 defects significantly enriched in this class as its phenotypic signature. Among those defects, *P_1_/AB asynchrony of division* and *four-cell stage configuration* are the characteristic defects of asymmetric cell divisions. Defects in *P_0_ pronuclear meeting*, *P_0_ spindle positioning*, *P_0_ spindle poles*, *P_1_ nuclear migration/rotation*, and *AB spindle orientation* are the ones that are likely to accompany the loss of asymmetry. We searched the rest of the dataset for additional genes with phenotypic profiles matching the signature (see Methods) and identified RGA-3, a putative Rho GTPase activating protein. This gene was originally classified as involved in *cortical structure*. Our search for phenotypic signatures did not rule out its functional involvement in cortical structure, but suggested its additional roles in cell polarity. A recent paper reported that knocking down RGA-3 along with its paralog RGA-4 resulted in changes in the boundary of anterior and posterior domains of PAR proteins in the early embryo [11]. This experiment confirmed our prediction for RGA-3's involvement in cell polarity. Such functional assignment of genes based on phenotypes may seem obvious, since genes sharing similar phenotypes should share similar functions. However, without the in-depth analysis of phenotypic signatures, additional roles of the genes are often neglected.

**Figure 6:**
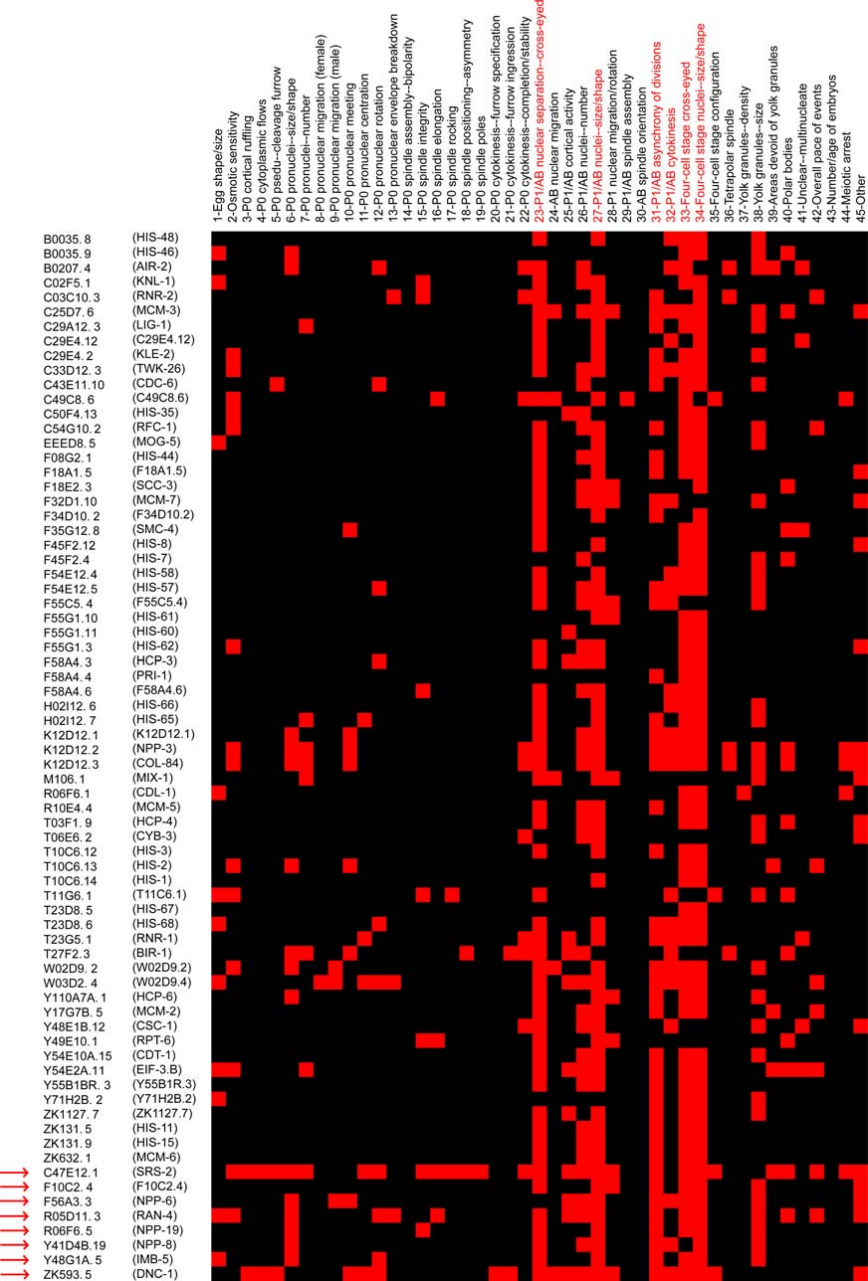
The phenotypic signature for the *chromosome function* class. This signature consists of 6 defects (highlighted in red). Eight additional genes (pointed to by red arrows) are assigned to the *chromosome function* class.

Another example of phenotypic signature is shown for the *chromosome function* class, which is a relatively large class consisting of 64 genes originally. Its phenotypic signature included *P_1_/AB nuclear separation—cross-eyed*, *P_1_/AB nuclei—size/shape*, *four-cell stage cross-eyed*, *four-cell stage nuclei—size/shape*, and so on (Figure 6). Using the phenotypic signature, we identified 8 additional genes for this class. The phenotypic profiles of these 8 genes all contain defects other than those included in the *chromosome function* signature, and thus were originally assigned to other classes. Interestingly, 5 of these 8 genes are known to be involved in nuclear transport functions, suggesting potential connections between nuclear transport and chromosome functions. Evidence supporting their roles in chromosome function has been reported in recent literature. NPP-8, which is part of the nuclear pore complex, was found to be recruited to the chromatin after anaphase onset in the early embryo [12]. NPP-19, another nuclear pore complex protein, along with F10C2.4, an uncharacterized gene, were both found to be tightly co-expressed with a group of genes involved in chromosome maintenance [13].

### Pleiotropic Genes

**Figure 7:**
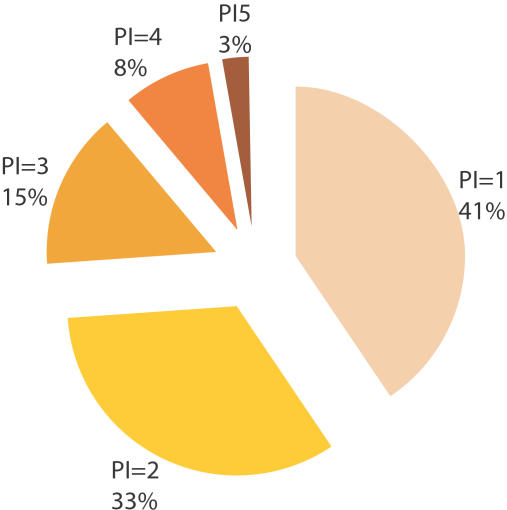
Distribution of pleiotropy indices. We define Pleiotropy Index (PI) as the number of functional classes a gene is assigned to. We plot the distribution of genes with different PIs in a pie chart. We observe that more than half of the genes are pleiotropic (PI≥2), and only 3% of the genes are highly pleiotropic (PI≥5).

By determining phenotypic signatures and identifying additional genes as belonging to each functional class, we allow genes playing multiple roles in early embryogenesis to be assigned to multiple classes. We define Pleiotropy Index as the number of classes a gene is assigned to. More than half of the genes involved in early embryogenesis are pleiotropic (i.e., with Pleiotropy Index ≥2), suggesting that pleiotropy occurs extensively (Figure 7). Genes that were not assigned to a functional class in the original screen are mostly pleiotropic (Table S1). Although the profiles of these genes do not resemble those of any other known genes, they now can be decomposed into several phenotypic signatures that lead to functional discoveries. For example, F25H2.4, an uncharacterized gene, is assigned to the classes of *cytoplasmic structure*, *mitochondrial function*, *meiotic cell cycle progression*, and *meiosis chromosome segregation*.

Although pleiotropy is relatively common, only 3% of the genes involved in early embryogenesis are highly pleiotropic (i.e., with Pleiotropy Index ≥5). Many signaling proteins show a very high Pleiotropy Index (Table S2), probably because signaling proteins can be part of various molecular machines functioning in early embryogenesis. For example, of all the 19 kinases involved in early embryogenesis, 18 are pleiotropic (95% compared to 59% of all genes involved in early embryogenesis), and 5 are highly pleiotropic (26% compared to 3% of all genes). The biochemical reaction that kinases catalyze is phosphorylation, and a single kinase can catalyze phosphorylation in multiple contexts and with different protein targets. Eliminating a kinase may thus result in multiple sets of defects because a variety of protein targets in different contexts cannot be phosphorylated properly.

Since the defects in consideration are not independent of each other, it is possible that the foregoing definition of Pleiotropy Index, although biologically meaningful, can be biased. To resolve this issue, we take the top 33 principal components (PCs) of the data matrix, which can account for 90% of the total variation, and regard them as “mega-defects.” Then, for a gene G, we define its influence from a functional class K as the average of the correlations of this gene's loading vector with those of all the genes in this class (see Methods). A gene G's Relative Pleiotropy Score is the sum of its influences from all functional classes. The Relative Pleiotropy Score does not have direct functional implications as Pleiotropy Index does, but it gives a relative value of how complex a phenotypic profile is and avoids over-counting highly correlated defects. We observe that the Relative Pleiotropy Score such defined is highly correlated with Pleiotropy Index (Figure S1), indicating that both are reasonable proxies to the concept of pleiotropy.

### Network Property of Highly Pleiotropic Genes

**Figure 8:**
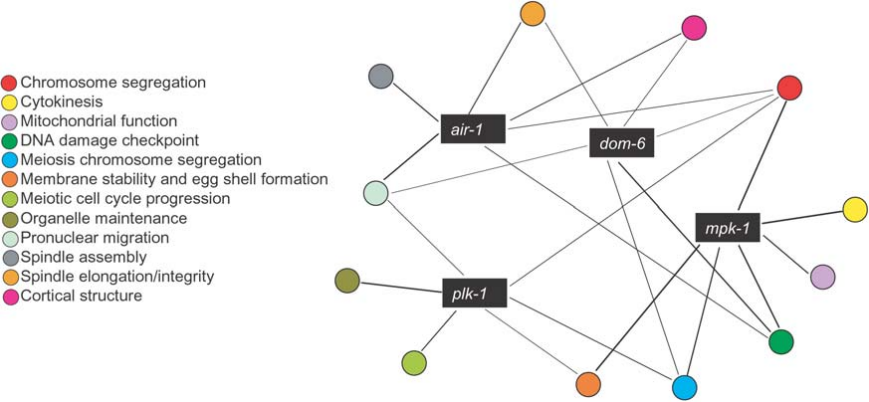
Pleiotropic genes as “module connectors”. The extensive existence of pleiotropic genes suggests that gene modules are overlapping rather than separate from one another. Genes assigned to the same functional class are represented as a module and pleiotropic genes correspond to the intersections of modules. In the illustrated example, the most pleiotropic kinases, including *dom-6*, *mpk-1*, *plk-1* and *air-1*, connect most of the modules in early embryogenesis into a “module network”.

Recent work has revealed a modular organization of genes and proteins in model organisms [13]–[18]. Here a module refers to a group of genes or proteins acting in concert to achieve a certain biological function. However, it is not yet clear how these modules are connected and coordinated. An immediate implication from our finding of pleiotropic genes is that gene modules overlap instead of being separate from one another. We hypothesized that pleiotropic genes act as “connectors” between different modules. The few most highly pleiotropic kinases, for instance, connect most of the major modules in early embryogenesis (Figure 8).

**Figure 9:**
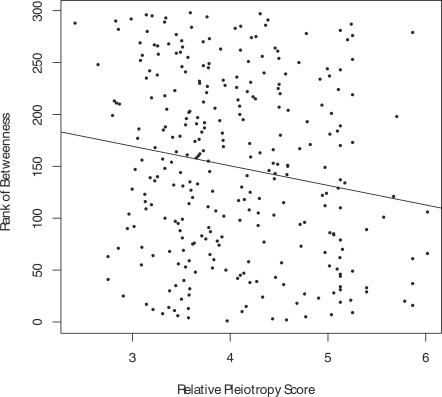
Scatter plot of Relative Pleiotropy Score and rank of betweenness. The rank of betweenness is significantly correlated with the Relative Pleiotropy Score. This correlation is largely contributed by the highly pleiotropic genes (upper right corner).

Many cellular events in early development are mediated by protein-protein interactions (PPIs). Complexes or pathways in PPI networks can be the molecular identities of modules. According to our hypothesis, the highly pleiotropic proteins we have identified should reside in central positions in the *C. elegans* PPI network [13],[19]. We tested our hypothesis by studying the relationship between a protein's “betweenness” and its Relative Pleiotropy Score or Pleiotropy Index. The betweenness of a given node is defined as the number of times that node is on the shortest paths connecting any two nodes in a network [9] (see Methods). It is a network property that measures the extent to which a node is topologically in a central position between sub-graphs of a network [9], and it has been applied to characterize modularity of biological networks [20],[21]. We ranked the betweenness values for early embryogenesis genes that involve two or more interactions in the network, and found that the rank of betweenness is significantly correlated with the Relative Pleiotropy Score (*P*-value = 0.004) (Figure 9). Furthermore, this statistical significance of the correlation appears to be contributed mostly by a few genes with the highest Relative Pleiotropy Scores. For example, the sum of betweenness ranks for the 12 genes with the highest Relative Pleiotropy Scores is 1123, whereas the sum of betweenness ranks for 12 randomly sampled early embryogenesis genes is 1794 on average (*P*-value = 0.01). Similarly, we found that the sum of betweenness values for the 11 genes with the highest Pleiotropy Indices (Pleiotropy Index≥5) is significantly higher than that for 11 early embryogenesis genes chosen at random (454701 vs. an average of 179400, *P*-value = 0.03) (see Methods). The betweenness property of highly pleiotropic genes presents supporting evidence to our hypothesis that pleiotropic genes act more as connectors between gene modules.

## Discussion

In this paper, we presented the first systematic investigation of pleiotropic genes in a multi-cellular organism. Using pre-defined functional classes as seeds, we identified phenotypic signatures associated with these classes, and then assigned genes based on their matches to the signatures. We annotated many uncharacterized genes with complex phenotypic profiles by decomposing their profiles into signatures that are indicative of biological functions. We also identified additional functions which were previously unknown for some characterized genes.

Our approach can potentially be generalized and applied to many other phenotypic datasets. For example, Gene Ontology categories can be used in place of pre-defined functional classes in order to obtain phenotypic signatures. Furthermore, the reproducibility of detecting defects in RNAi experiments may also be used to define signatures from large amount of phenotypic profiles.

Although each gene identified as required for early embryogenesis was assigned to only one class in the original RNAi screen, we found that nearly half of these genes are pleiotropic. Some genes, in particular those encoding signaling molecules, are highly pleiotropic. We examined evolutionary rates of highly pleiotropic genes by comparing sequences from *C. elegans* and *C. briggsae*. We found that highly pleiotropic genes evolved at similar rates to other early embryogenesis genes (data not shown), suggesting that pleiotropy may not constitute severe constraints for protein evolution. Our finding is consistent with a previous report that pleiotropic and non-pleiotropic genes evolve at similar rates in yeast [22]. We also assessed the possibility that abundantly expressed genes are more likely to be highly pleiotropic. We retrieved the expression levels of early embryogenesis genes from a SAGE (Serial Analysis of Gene Expression) dataset [23], and correlated with Pleiotropy Index. By performing linear regression we found a significant negative correlation between expression level and Pleiotropy Index (*P*-value<0.01) (Figure S2). The highly pleiotropic genes tend to be less abundantly expressed than genes assigned with only one or two phenotypic signatures. This is consistent with our observation that signaling molecules such as kinases are enriched in the set of highly pleiotropic genes. The genes involved in cell signaling are often only expressed at a low level but play very important regulatory roles.

Finally, we proposed a mechanistic interpretation of pleiotropy from the perspective of functional modules in cellular networks. Since pleiotropic genes are multi-functional, we reasoned that they are likely to coordinate distinct functions involved in early embryogenesis. Consistent with this notion, we found that highly pleiotropic genes exhibit higher betweenness in PPI networks than randomly selected genes. However, there are examples of non-pleiotropic genes showing high betweenness and high pleiotropic genes showing low betweenness. A potential reason is that current PPI data is neither comprehensive nor precise. False positives and false negatives exist in the datasets of genome-wide yeast two-hybrid screens. Consequently, the estimation of centrality based on betweenness may not accurate for every protein in the network. Another possible reason is that mechanisms other than centrality in PPI networks may contribute to pleiotropy. Hodgkin discussed possible underlying mechanisms of pleiotropy and classified them into seven different types [24]. “Combinatorial pleiotropy”, the situation that a protein plays various roles through its various binding partners, is only one type of mechanism. This mechanism is important for the pleiotropy in early embryogenesis, probably because many protein complexes mediate this process.

It is not clear yet what mechanisms underlie pleiotropy in other biological processes in multi-cellular organisms. We combined results from two genome-wide RNAi screens [25],[26] which scored maternal sterility, embryonic lethality, and a limited number of post-embryonic defects with the *C. elegans* PPI networks. We found 7 genes that exhibited 8 or more of the scored defects and had 2 or more interactions. These 7 genes had a higher sum of betweenness values than that of 7 randomly selected genes, though the *P*-value of the difference is marginal (*P*-value = 0.09). This result indicates that PPI networks may contribute to pleiotropy in a broader context, but other mechanisms of pleiotropy probably apply as well. Currently, few datasets that score a large number of phenotypes in detail are available for multi-cellular organisms. The mechanisms underlying pleiotropy are worth further investigations once we have more comprehensive and accurate phenotypic profiles as well as other types of functional genomic data.

## Methods

### Permutation of Genes and Their Loss-of-Function Defects

Phenotypic profiles were represented as a binary matrix where rows indexed genes and columns indexed defects. Each entry in the matrix was either zero or a positive number, indicating the absence or presence of defects. We obtained control datasets by randomly permuting values among genes for each column while keeping the number of positive cells in each column fixed.

### Co-occurrence of Defects and the Construction of a Correlation Map

We calculated the frequency of occurrence for each individual defect (*F*(*i*)) and the frequency of co-occurrence for each pair-wise combination of defects (*F*(*i*,*j*)).
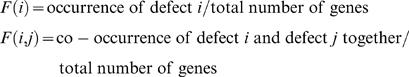



For each pair of defects, we calculated the ratio (*R*(*i*,*j*)) of the observed co-occurrence frequency over the expected frequency as if the two defects occurred independently: R(*i*,*j*) = F(*i*,*j*)/(F(*i*)×F(*j*)). We generated a map of R(*i*,*j*) using the heatmap function in the statistical language R.

### Enrichment of Defects in Functional Classes

There were 22 manually assigned functional classes in the phenotypic dataset. We used genes originally assigned in a class as seeds to identify defects enriched in that class. The collection of enriched defects was defined as the phenotypic signature of the given class. We used the cumulative hypergeometric distribution to determine whether a defect was significantly enriched in a class compared to the whole dataset. In a given class, if the phenotypic profiles of *x* genes contained a given defect, the *P*-value was calculated as the following:
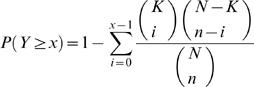
In this formula, *N* represents the total number of genes in the dataset; *K* represents the total number of genes for which phenotypic profiles contain the given defects; *n* represents the number of genes in the given class.

### Matching Phenotypic Profiles to Phenotypic Signatures

For each functional class, we examined whether any additional genes can be assigned to the given class by matching phenotypic profiles to the identified signature of that class. First, we obtained phenotypic profiles of genes originally assigned to the given class and calculated the average number (“A”) of defects matching the signature of that class. Second, we obtained phenotypic profiles of genes not originally belonging to that class and scored them by the number of defects matching the signature. If a gene scored equal to or higher than A, this gene was assigned to the given class. This procedure does not require a perfect match, but it does make the enrichment of defects in the signatures even more enriched in each individual class. In the procedure, we allowed genes to be assigned to multiple classes besides their original assignment, since some genes might play more than one role in early embryogenesis.

Phenotypic signatures of different classes contain different sets of defects. In a few cases, the signature of one class (*X*) contains all the defects from the signature of another class (*Y*). In other words, the defects in the signature of class *Y* are a subset of that of class *X*. Thus, a phenotypic profile containing all the defects of the signature for class *X* automatically contains all the defects of the signature for class *Y*. In order not to overestimate the degree of pleiotropy, genes with phenotypic profiles matching the signature of *X* are only assigned to class *X*, instead of both *X* and *Y*. For example, the signature of the *protein synthesis* class contains all of the defects from the signatures of the *cytoplasmic structure*, *meiosis chromosome segregation*, *chromosome segregation*, and *mitochondrial function* classes. It can be speculated that blocking protein synthesis results in a number of deleterious effects that resemble perturbing cytoplasmic structure, meiosis chromosome segregation, chromosome segregation, and mitochondrial functions. Thus genes assigned to the *protein synthesis* class were not considered for assignment to any of the above classes.

### LPCA Analysis of Phenotypic Data

LPCA is a dimensionality reduction method for binary data [10]. We applied LPCA to the phenotypic profiles of early embryogenesis genes and projected all the defects onto the first two principal components for visualization. The MATLAB code of LPCA was downloaded from www.cis.upenn.edu/∼ais/software/lpca_code.tar.

### Principal Component Analysis and Relative Pleiotropy Scores

We applied PCA to the phenotypic profiles which consist of 661 genes in rows and 45 defects in columns. Eigenvalue diagnosis indicated that 33 principle components accounted for 90% of the variation in the dataset. We calculated an average of Pearson correlation coefficients between the gene of interest and any genes from a given functional class. The relative pleiotropy score is defined as the sum of average Pearson correlation coefficients of all the functional classes.

### Calculation and Comparison of Betweenness

The betweenness of a node is defined as the number of shortest paths running through the node of interest [9]. We computed the shortest paths between all pairs of nodes in the largest component of *C. elegans* PPI networks [13],[19]. For each pair of nodes, we enumerated all possible paths in between the chosen pair and increased the betweenness score of the nodes on the shortest paths by one. If there were N alternative shortest paths on route, we split the credit and assigned partial score 1/N to the nodes on the shortest paths. We computed betweenness values for proteins that interact with at least two other proteins, because a protein with only one interacting partner could not be on any shortest paths except for the paths involving the protein itself. We calculated the sum of betweenness values for the early embryogenesis proteins with Pleiotropy Index of 5 or higher. The *P*-value of significance was estimated by randomly selecting the same number of early embryogenesis genes that had betweenness values and by calculating the sum of their betweenness values. The simulation was repeated 1,000,000 times.

## Supporting Information

Figure S1 A scatter plot of the Pleiotropy Index and the Relative Pleiotropy Score. These two measures are significantly correlated.
(0.03 MB DOC)

Figure S2 A scatter plot of the Pleiotropy Index and the expression level measured in a SAGE dataset. Genes with Pleiotropy Index equal or greater than 5 are grouped together.
(0.03 MB DOC)

Table S1 Functional annotation of genes with complex phenotypic profiles.
(0.02 MB DOC)

Table S2 Highly pleiotropic genes.
(0.02 MB DOC)
