# Supplementary material for: Systematic Analysis of Pleiotropy in C. elegans Early Embryogenesis
Source: PLoS Comput Biol. 2008 Feb 29;4(2):e1000003. doi: 10.1371/journal.pcbi.1000003 (PMC2265476; doi:10.1371/journal.pcbi.1000003)

Figure S1. A scatter plot of the Pleiotropy Index and the Relative Pleiotropy Score. These

two measures are significantly correlated.


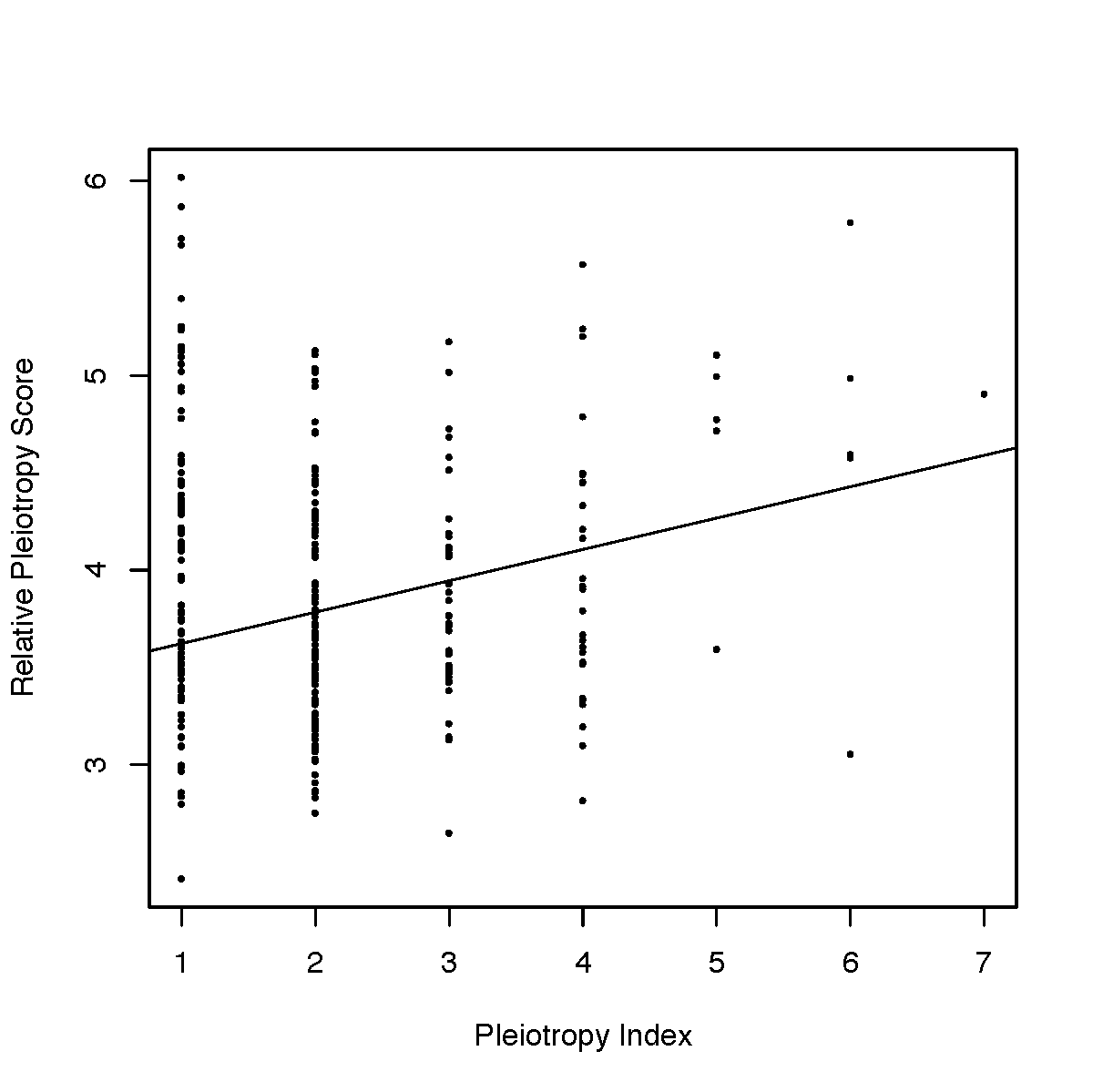

Supplement: Figure S1 — A scatter plot of the Pleiotropy Index and the Relative Pleiotropy Score. These two measures are significantly correlated. (0.03 MB DOC) [file pcbi.1000003.s001.doc]
