# Supplementary material for: Systematic Analysis of Pleiotropy in C. elegans Early Embryogenesis
Source: PLoS Comput Biol. 2008 Feb 29;4(2):e1000003. doi: 10.1371/journal.pcbi.1000003 (PMC2265476; doi:10.1371/journal.pcbi.1000003)
Supplement: Figure S2 — A scatter plot of the Pleiotropy Index and the expression level measured in a SAGE dataset. Genes with Pleiotropy Index equal or greater than 5 are grouped together. (0.03 MB DOC) [file pcbi.1000003.s002.doc]

Figure S2. A scatter plot of the Pleiotropy Index and the expression level measured in a

SAGE dataset. Genes with Pleiotropy Index equal or greater than 5 are grouped together.
