# Supplementary material for: Systematic Analysis of Pleiotropy in C. elegans Early Embryogenesis
Source: PLoS Comput Biol. 2008 Feb 29;4(2):e1000003. doi: 10.1371/journal.pcbi.1000003 (PMC2265476; doi:10.1371/journal.pcbi.1000003)
Supplement: Table S1 — Functional annotation of genes with complex phenotypic profiles. (0.02 MB DOC) [file pcbi.1000003.s003.doc]

Table S1. Functional annotation of genes with complex phenotypic profiles.

| **Gene** | **Functional Annotation** |
| --- | --- |
| C14B9.4 (*plk-1*) | chromosome segregation, organelle maintenance, membrane stability, meiotic cell cycle progression, meiosis chromosome segregation, pronuclear migration |
| F14B8.1 (*nhx-4*) | cytoplasmic structure, cortical structure, mitochondrial function, organelle maintenance, membrane stability, spindle elongation/integrity |
| F43C1.2 (*mpk-1*) | chromosome segregation, cytokinesis, mitochondrial function, membrane stability, DNA damage checkpoint, meiosis chromosome segregation |
| Y75B8A.30 (*pph-4.1*) | cytoplasmic structure, chromosome segregation, cortical structure, nuclei reformation, spindle assembly |
| C08B11.1 (*zyg-11*) | cortical structure, meiosis progression, nuclei reformation, protein synthesis |
| F25H2.4 | cytoplasmic structure, mitochondrial function, meiosis cell cycle progression, meiosis chromosome segregation |
| F26H9.6 (*rab-5*) | chromosome segregation, mitochondrial function, organelle maintenance, nuclei reformation |
| H38K22.2 | chromosome segregation, cytokinesis, DNA damage checkpoint, pronuclear migration |
| Y71H2B.3 | cortical structure, pronuclear migration, nuclei reformation, protein synthesis |
| M7.1 (*let-70*) | cytoplasmic structure, cortical structure, mitochondrial function, meiotic cell cycle progression, |
| C18E9.6 | cytoplasmic structure, chromosome segregation, DNA damage checkpoint, |
| C39B5.2 | meiotic cell cycle progression, meiosis chromosome segregation, spindle elongation/integrity |
| F45H11.2 (*ned-8*) | chromosome segregation, DNA damage checkpoint, meiosis chromosome segregation |
| Y37B11A.3 | chromosome segregation, cortical structure, meiosis chromosome segregation |
| Y82E9BR.15 (*elc-1*) | cortical structure, pronuclear migration, protein synthesis |
| F07A5.1 (*inx-14*) | meiotic cell cycle progression, protein synthesis |
| Y18D10A.17 | chromosome segregation, cortical structure |
