# Supplementary material for: Systematic Analysis of Pleiotropy in C. elegans Early Embryogenesis
Source: PLoS Comput Biol. 2008 Feb 29;4(2):e1000003. doi: 10.1371/journal.pcbi.1000003 (PMC2265476; doi:10.1371/journal.pcbi.1000003)
Supplement: Table S2 — Highly pleiotropic genes. (0.02 MB DOC) [file pcbi.1000003.s004.doc]

Table S2. Highly pleiotropic genes.

| **Gene** | **Description** | **Pleiotropy index** |
| --- | --- | --- |
| C09G4.3 (*dom-6*) | cyclin-dependent kinase regulatory subunit | 7 |
| C47E12.1 (*srs-2*) | seryl-tRNA synthetase | 7 |
| Y43E12A.1 (*cyb-2.1*) | cyclin B | 6 |
| C14B9.4 (*plk-1*) | polo-like serine/threonine kinase | 6 |
| F14B8.1 (*nhx-4*) | sodium/proton exchanger | 6 |
| F43C1.2 (*mpk-1*) | mitogen-activated protein (MAP) kinase | 6 |
| K07C11.2 (*air-1*) | Aurora-A family of serine/threonine kinase | 6 |
| K09H11.3 | Rho GTPase activating protein | 6 |
| Y55F3AM.15 (*csn-4*) | protein with similarity to human COP9 proteasome subunit 4 | 6 |
| C01H6.5 (*nhr-23*) | nuclear hormone receptor | 5 |
| F32H2.3 (*spd-2*) | protein with coiled-coil domains, required for centrosome maturation and duplication | 5 |
| H37A05.1 | N/A | 5 |
| T06G6.9 (*vbp-1*) | human VHL binding protein like | 5 |
| T26A5.9 (*dlc-1*) | dynein light chain type 1 | 5 |
| Y74C10AR.1 (*eif-3.I*) | translation initiation factor 3, subunit i | 5 |
| Y75B8A.30 (*pph-4.1*) | serine/theorine protein phosphatase | 5 |
| ZC204.11 | N/A | 5 |
| D2030.4 | NADH:CoQ oxidoreductase subunit B18 | 5 |
| F28B12.3 | protein kinase | 5 |
